# A Hypothetical Approach to Concentrate Microorganisms from Human Urine Samples Using Paper-Based Adsorbents for Point-of-Care Molecular Assays

**DOI:** 10.3390/life14010038

**Published:** 2023-12-25

**Authors:** Isha Uttam, Sujesh Sudarsan, Rohitraj Ray, Raja Chinnappan, Ahmed Yaqinuddin, Khaled Al-Kattan, Naresh Kumar Mani

**Affiliations:** 1 Microfluidics, Sensors and Diagnostics (µSenD) Laboratory, Centre for Microfluidics, Biomarkers, Photoceutics and Sensors (μBioPS), Department of Biotechnology, Manipal Institute of Technology, Manipal Academy of Higher Education, Manipal 576104, Karnataka, India; ishauttam2003@gmail.com (I.U.); sujeshmayyil325@gmail.com (S.S.); 2 Department of BioSystems Science and Engineering (BSSE), Indian Institute of Science, CV Raman Rd, Bangalore 560012, Karnataka, India; 9980rohitray@gmail.com; 3 College of Medicine, Alfaisal University, Riyadh 11533, Saudi Arabia; ayaqinuddin@alfaisal.edu (A.Y.); kkattan@alfaisal.edu (K.A.-K.); 4 Tissue/Organ Bioengineering & BioMEMS Lab, Organ Transplant Centre of Excellence, Transplant Research & Innovation Department, King Faisal Specialist Hospital and Research Centre, Riyadh 11211, Saudi Arabia

**Keywords:** preconcentration, loop-mediated isothermal amplification (LAMP), urine, microbial adhesion, hydrophobic, paper-based analytical devices (PADs), cell surface hydrophobicity (CSH), UTI

## Abstract

This hypothesis demonstrates that the efficiency of loop-mediated isothermal amplification (LAMP) for nucleic acid detection can be positively influenced by the preconcentration of microbial cells onto hydrophobic paper surfaces. The mechanism of this model is based on the high affinity of microbes towards hydrophobic surfaces. Extensive studies have demonstrated that hydrophobic surfaces exhibit enhanced bacterial and fungal adhesion. By exploiting this inherent affinity of hydrophobic paper substrates, the preconcentration approach enables the adherence of a greater number of target cells, resulting in a higher concentration of target templates for amplification directly from urine samples. In contrast to conventional methods, which often involve complex procedures, this approach offers a simpler, cost-effective, and user-friendly alternative. Moreover, the integration of cell adhesion, LAMP amplification, and signal readout within paper origami-based devices can provide a portable, robust, and highly efficient platform for rapid nucleic acid detection. This innovative hypothesis holds significant potential for point-of-care (POC) diagnostics and field surveillance applications. Further research and development in this field will advance the implementation of this technology, contributing to improved healthcare systems and public health outcomes.

## 1. Introduction

The human body acts as a favorable environment for pathogenic organisms to invade, grow, and reproduce. Our body comprises cells in the order of 10^13^ as well as thousands of microbial species, containing approximately 10^14^–10^15^ bacterial cells [1]. The microbial colonization in distinct regions of the human body, specifically the skin, oral cavity, gastrointestinal tract, and vaginal mucosa, exerts a profound impact on the development of a wide range of infections [2]. Notably, urinary tract infection (UTI) emerges as a pervasive and clinically significant infection among them [3,4]. It is worth highlighting that around 50% of individuals have encountered the distressing experience of a UTI at least once in their lifetime [5]. Also, it is crucial to acknowledge the significant financial burden imposed by UTIs, as evidenced by an expenditure of nearly USD 2.8 billion in 2011 [6]. The etiology of these infections involves a diverse range of microbial agents, encompassing Gram-positive bacteria, Gram-negative bacteria, and fungi [7].

The uropathogenic *Escherichia coli*, a member of the Enterobacteriaceae family, stands out as the primary causative agent, accounting for more than 80% of UTI cases [3]. Other pathogens include *Klebsiella* species, *Proteus* species, *Pseudomonas aeruginosa*, and *Enterococcus* species [8]. These infections exert a substantial impact on the morbidity rates of different patient populations, including infants, elderly males, and females of all age groups [7]. UTI is divided into two categories based on disease progression among vulnerable individuals. The first category is known as “uncomplicated UTI”, which primarily affects individuals without underlying health conditions. The second category is called “complicated UTI”, which is associated with factors such as catheterization, immunosuppression, or prior antibiotic usage [9]. Technically, for a patient presenting symptoms of a urinary tract infection, any microbial concentration in the test results may indicate an infection. However, in order to be clinically diagnosed as a UTI, the microbial load should surpass a threshold of 10^5^ colony-forming units per milliliter (CFU/mL) of urine [10]. In the realm of urinary tract infection detection, the gold standard diagnostic method involves the collection of urine samples, followed by a subsequent culture [11]. Although this approach is highly reliable, it requires a turnaround time of 2–3 days for result acquisition. Additionally, diagnostic methods used in well-developed regions demand advanced laboratory facilities and expensive chemicals. Consequently, these methods are unsuitable for areas with limited financial means, rudimentary healthcare infrastructure, and a scarcity of adequately trained personnel [12]. Commercial urinary-tract-infection dipstick tests are available as an alternative; however, their results are less reliable compared to urine culture tests [7].

Recently, there has been a growing interest and importance surrounding microfluidic paper-based analytical devices (µPADs) and microfluidic thread-based analytical devices (µTADs) for the detection of pathogen biomarkers [13,14,15,16,17,18,19,20], food adulterants [21,22,23], and chemical analyte detection [24]. These devices focus on the unique properties of paper such as uniform thickness, high hygroscopicity, optimal wicking, desirable flow rates, consistent fluid flow, efficient sample absorption, and reliable analyte transportation [25]. Furthermore, paper can be easily modified and functionalized with specific chemicals or coatings, allowing for selective detection of disease-specific biomarkers or pathogens through visual color changes or other readout methods [24]. The World Health Organization (WHO) has established essential guidelines for future diagnostics through the ASSURED criteria, which stands for affordable, sensitive, specific, user-friendly, rapid, and robust, equipment-free and deliverable to end users [12]. Point-of-care (POC) diagnostics offer a promising solution that aligns with these defined criteria, presenting numerous advantages such as affordability, sustainability, portability, disposability, simplicity, and the ability to handle very small volumes of samples [12]. The simplicity and robustness of µPADs facilitate effortless operation without the need for extensive training [26]. Furthermore, their cost-effective manufacturing renders them well-suited for large-scale production, making them accessible to a wide population who may not have the means to afford expensive and highly sophisticated diagnostic alternatives [27]. They also eliminate the need for complex machinery, making them ideal for clinical use [12].

**Figure 1:**
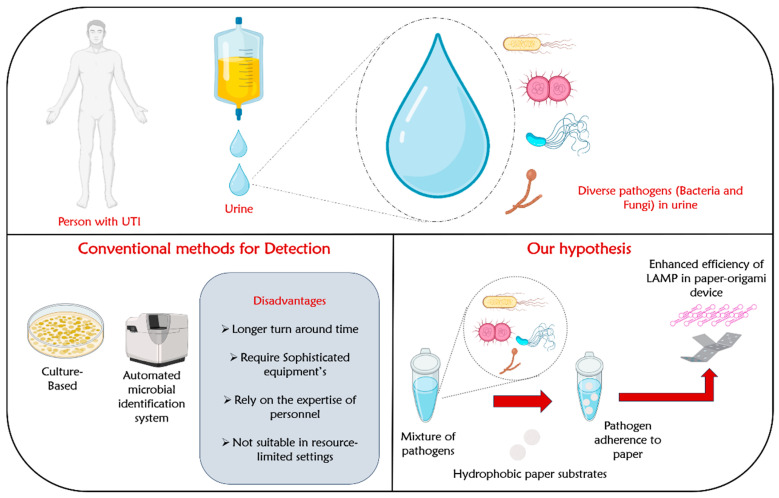
Schematic illustration showing dynamic paper substrates with tuned hydrophobicity for capturing pathogens from human urine samples.

In the past few years, the benefits of loop-mediated isothermal amplification (LAMP) on microfluidic paper-based analytical devices (µPADs) are opening new avenues for point-of-care nucleic acid testing in resource-limited settings [28]. The LAMP process amplifies samples at a constant temperature by repeatedly undergoing two distinct elongation reactions within the loop regions. These reactions involve the self-extension of templates originating from the stem loop structure formed at the 3′-terminal and the binding and extension of new primers within the loop region. The LAMP technique holds significant potential for a variety of uses, including point-of-care diagnostics, genetic testing in regions with limited resources (like developing countries), and swift testing of food items and environmental samples [28]. LAMP can be considered as a low-cost alternative to PCR. It is highly specific due to the involvement of five distinct primers [28]. Furthermore, this method is easy to use, as it enables the amplification to be carried out in a dry block heater or an incubator. Despite its numerous advantages, LAMP occasionally exhibits reduced sensitivity, necessitating a range of reaction additives and enhancement strategies to improve its sensitivity [29]. These strategies include the use of crowding agents, stabilizing nucleic acid structures, enzyme stabilization, oligonucleotide modifications, and template blockers [29]. However, compared to the simple and facile approaches to increase the amplification efficiency, these strategies have several inherent disadvantages. Firstly, they add complexity to the LAMP protocol, involving additional steps, reagents, and optimization, which complicate the workflow and increase the chance of errors. Secondly, the use of sensitivity enhancement techniques often leads to higher costs due to the need for specialized and potentially expensive reagents and modifications, making them less accessible in resource-limited settings. A schematic illustration showing dynamic paper substrates with tuned hydrophobicity for capturing pathogens from human urine samples is shown in Figure 1.

In light of the limitations mentioned above, our hypothesis aims to explore the potential of preconcentrating microbial cells onto paper surfaces as a means of enhancing the efficiency of LAMP amplification. Generally, different pathogens, especially bacteria, exhibit varying degrees of binding affinities to abiotic surfaces based on their diverse hydrophobic characteristics [30]. Also, it has been demonstrated that surfaces with intermediate wettability (hydrophobic) demonstrate a higher affinity for bacterial or cellular binding compared to surfaces that are extremely hydrophobic or hydrophilic [31]. A study by Yuan et al. demonstrated the presence of trapped air at the interface of superhydrophobic surfaces, which prevented bacteria from directly coming into contact with solid surfaces, resulting in decreased bacterial adhesion. In the case of the superhydrophobic surface with a lower solid area fraction, it exhibited a self-cleaning capability by effectively removing initially adhered bacteria during the washing process. On the other hand, a super hydrophilic substrate with a negative zeta potential displayed minimal bacterial attachment, primarily due to the reduced hydrophobic interaction and the potential repulsion between bacteria and the surface [31]. Building upon these compelling observations, we formulate the hypothesis that most of the pathogens in the urine sample would adhere to hydrophobic paper-based devices compared to its hydrophilic or superhydrophobic counterparts. By leveraging this characteristic, we can facilitate the concentration of microbial cells onto hydrophobic paper surfaces, thereby improving efficiency of the subsequent amplification, and resulting in specific pathogen detection.

## 2. The Hypotheses

### 2.1. Paper-Based Adsorbents as Potential Microbial Concentration Media from Human Urine Samples

The majority of conventional methods and biosensors employed for detecting disease-causing microorganisms rely on pre-concentration methods to enhance their efficiency. Several preconcentration methods such as liquid-liquid extraction (LLE), solid-phase extraction (SPE), solid-phase microextraction (SPME) [32], point extraction [33], and magnetic isolation [34,35] are used for preconcentrating the analytes in the biological fluids before analysis. Though these techniques are efficient and reliable, they have limitations such as time-consuming procedures, the need for specialized laboratory equipment, high cost, etc. Thus, our hypothesis deals with a robust method of using disposable paper-based adsorbents to selectively concentrate microbial cells present in the urine sample. The device works based on the interaction between the hydrophobic paper surface and the cell surface of the microorganisms. In this model, we exploited the concept of cell surface hydrophobicity (CSH) exhibited by pathogens to effectively concentrate the cells from the urine samples. Drawing insights from the existing literature, we observed that bacterial and fungal pathogens generally display a higher affinity for binding towards moderately hydrophobic surfaces, having a water contact angle of 90° for adhesion [31]. Paper is a versatile material that can be easily tuned to produce these hydrophobic surfaces. We anticipate that the dynamic interaction between the pathogenic cells and the hydrophobic paper will facilitate the selective adhesion process, resulting in effective cell concentration from the sample. When delving into the discussion on the selective adsorption of pathogens, it is essential to address the aspect of non-selective adsorption of metabolic waste products/residual impurities in urine. Generally, the major metabolic waste products in urine are urea, creatinine, ammonia, uric acid, and inorganic salts [36,37]. Urea and creatinine being polar molecules, are inherently hydrophilic due to the presence of functional groups capable of forming hydrogen bonds [38]. Consequently, they are less likely to bind to hydrophobic surfaces. Similarly, ammonia (NH₃) is a polar molecule with a lone pair of electrons. Thus, hydrophilic surfaces, which attract water, may facilitate favorable interactions with these compounds [39]. Inorganic salts, which dissociate into ions in solution, generally exhibit hydrophilic behavior. As a result, the interaction of inorganic salts with hydrophobic surfaces is expected to be unfavorable [40]. The aforementioned chemical characteristics of these metabolic compounds in urine are advantageous in our proposed methodology, where the hydrophobic surface tends to selectively adsorb pathogens without adsorbing the rest of the impurities. Moreover, the inherent advantage of loop-mediated isothermal amplification (LAMP), specifically, its tolerance to amplification inhibitors, enables robust amplification even in the presence of minute quantities of impurities in urine. These features complement the strengths of our proposed methodology, enhancing its potential for accurate and reliable nucleic acid detection. Furthermore, investigating various surface modification techniques on hydrophobic paper surfaces offers insights into achieving the desired balance between pathogen adsorption and impurity exclusion. Techniques such as nanotexturing [41], functionalization with specific ligands, or the incorporation of nanostructures [40] could be explored to fine-tune the hydrophobic surface for selective pathogen capture.

Thus, the preconcentration method is invaluable for obtaining accurate results in both PCR and loop-mediated isothermal amplification (LAMP). Furthermore, the technique is advantageous in scenarios with a lower pathogen concentration during the early stage of infections.

### 2.2. Evaluation of the Hypotheses: Tuning the Hydrophobicity of Paper Surfaces for Preconcentrating the Microorganisms from Urine Samples

The interaction between microbes and abiotic surfaces is influenced by a range of factors, including surface charge density, roughness, topography, stiffness, and hydrophobicity [30]. Given that microbes, particularly bacteria, typically bear a net negative charge due to the presence of amino, carboxyl, and phosphate groups on their cell wall surfaces [42], positively charged surfaces are more conducive to enhanced microbial adhesion. In an initial exploration of the influence of surface charge on bacterial adhesion, Gottenbos et al. demonstrated that *Pseudomonas aeruginosa* exhibited a two-fold increase in both initial adhesion and growth on positively charged poly (methacrylates) in comparison to negatively charged surfaces [43]. Extensive research has been conducted to investigate the influence of surface roughness on bacterial adhesion. According to Yoda et al., an increase in surface roughness increases the available surface area for bacterial attachment, providing a platform for adhesion [44]. Moreover, rough surfaces safeguard bacteria from shear forces [30], consequently impeding their detachment. Hence, the prevailing consensus asserts that as surface roughness increases, bacterial adhesion also increases. It was observed by James et al. that *Staphylococcus epidermidis*, *Pseudomonas aeruginosa*, and *Ralstonia pickettii* exhibited significantly greater adhesion on surfaces characterized by increased roughness and larger surface areas, as opposed to smoother surfaces [45]. Furthermore, bacteria possess the ability to perceive mechanical signals linked to surface attributes, including surface topography. Observations have revealed that alterations in surface topography, known to affect the expression of bacterial adhesins, subsequently impact bacterial adsorption [30]. Stiffness is another important factor that affects bacterial adhesion. Kolewe et al. found that bacterial adhesion increases with increasing material stiffness regardless of the surface chemistry or adhesion mechanism [46].

Microbial adhesion to diverse surfaces, including the air/water interface, biomaterials, and various solid surfaces, is profoundly influenced by the pivotal factor of cell surface hydrophobicity (CSH), a biophysical measurement of a cell’s affinity for a hydrophobic versus hydrophilic environment [47]. Cells with greater surface hydrophobicity adhere strongly to hydrophobic surfaces, whereas hydrophilic cells exhibit robust adhesion to hydrophilic surfaces [48]. Rooted in this foundation, our postulate seeks to explore the potential of preconcentrating microbial cells in urine onto hydrophobic paper surfaces as a means of improving LAMP amplification efficiency. We draw upon the understanding that surfaces with intermediate wettability, specifically hydrophobicity, demonstrate a higher affinity for bacterial or cellular binding compared to extremely hydrophobic or hydrophilic surfaces [31]. The adhesive forces between a hydrophobic abiotic substrate and microbes arise through van der Waals and electrostatic double-layer interactions [49]. Numerous studies have documented comparable results and reported similar findings. For instance, Tegoulia and Cooper utilized thiol surfaces with differing functional end groups to study the effect of surface hydrophilicity and hydrophobicity on *Staphylococcus aureus* adhesion and found that the bacterial adhesion was higher on the hydrophobic surfaces [50].

**Figure 2:**
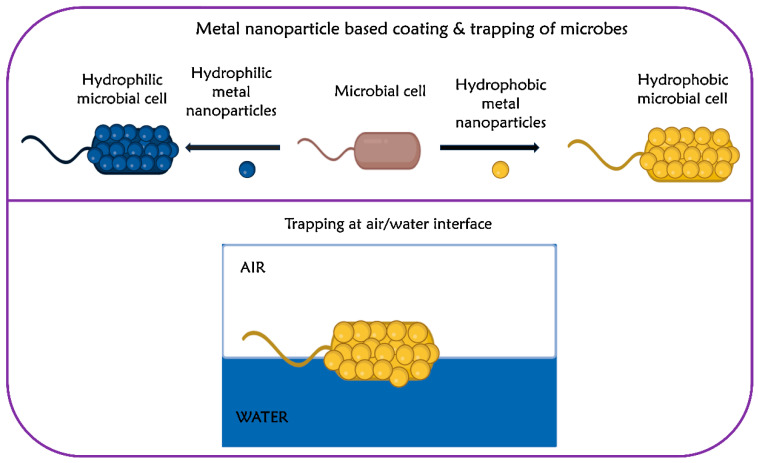
Modification of bacteria using metal nanoparticles and trapping them at the air/water interface. Adapted with permission from ref. [51].

Kim et al. introduced a novel approach to modify the surface of microbial cells using hydrophobic gold nanoparticles [51]. The findings revealed that *E. coli*, when coated with hydrophobic gold nanoparticles, displayed an irreversible entrapment at the air/water interface, owing to increased hydrophobicity (Figure 2) [51]. Based on the findings, the authors inferred that the broad applicability of the microbial surface transformation method and dynamic interfacial trapping could be possibly extended to other organisms including Gram-positive bacteria, Gram-negative bacteria, and fungi. As the dynamic interfacial trapping allows the preconcentration of microbial cells, the authors successfully quantified *E. coli* at concentrations as low as 1.0 × 10^5^ cells/mL, using a smartphone with an image analyzer [52]. A research study conducted by Yuan et al. demonstrated that moderate hydrophobicity with a water contact angle (WCA) of about 90° showed enhanced adhesion of *E. coli* on polymeric substrates, whereas the adhesion of the bacteria on hydrophilic surfaces and superhydrophobic surfaces was found to be limited [31]. The reduced affinity of the bacteria to hydrophilic surfaces was attributed to reduced hydrophobic interaction and the repulsive interaction between the bacteria and the substrate [31].

Conversely, when the bacteria were exposed to a superhydrophobic surface, the contact area fraction between the pathogen and the surface was reduced due to the air entrapment at the interface, leading to lower bacterial adhesion [31]. According to Hizal et al., the synergistic effect of superhydrophobic surfaces and fluid shear stress led to a decrease in the attachment of *Staphylococcus aureus* and *Escherichia coli*. This reduction was attributed to the slippery nature of the superhydrophobic surface [53]. Previously, the relationship between surface energy/interfacial interaction energy and bacterial adhesion has been elucidated by various models, including the thermodynamic theory [49]. According to this theory, bacteria with hydrophobic cell surfaces exhibit a preference for hydrophobic surfaces, which have a lower surface energy, while bacteria with hydrophilic cell surfaces tend to favor hydrophilic surfaces, which possess higher surface energy [48].

The adherence of pathogens onto hydrophobic surfaces is attributed to various underlying mechanisms that are specific to different pathogens. These mechanisms enable pathogens to interact and bind more effectively to hydrophobic surfaces. One contributing factor to pathogen adhesion on hydrophobic surfaces is the development of specific adaptive mechanisms for their survival [54]. These mechanisms enable bacteria to modify their cell surfaces in response to toxicity and limited availability of nutrients [54]. By adjusting their hydrophobicity, bacteria facilitate direct hydrophobic–hydrophobic interactions with the substrates, promoting adhesion [54]. In addition to these adaptations, some pathogens exhibit changes in their cell surface hydrophobicity (CSH) [48]. Microbial CSH can be influenced by several variables, including difference in cell wall composition, genetic modification and alteration in temperature. For instance, in *Aspergillus* spp., a protein called hydrophobins have different hydrophobic domains that allow interactions with hydrophobic surfaces [55]. Hydrophobins possess moderate to high hydrophobicity and have been extensively studied for their involvement in facilitating hydrophobic interactions [56]. In addition to that, other components of the cell wall including mannoproteins, glucans, and lipids can also contribute to CSH [57]. Table 1 provides a comprehensive overview of the common pathogens found in urine along with their favorable surface hydrophobicity for adhesion. A compilation of existing literature on microbial adhesion to various hydrophobic material surfaces is depicted in Table 2. The influence of different surface materials for microbial adhesion and biofilm formation is given in Table 3.

### 2.3. Evaluation of the Hypotheses: Does Preconcentrating the Microorganisms from Urine Samples Facilitate the Accuracy of LAMP and PCR?

Extensive research findings have unveiled the remarkable potential of paper substrates in achieving the precise tuning of hydrophobicity [55,67]. This arises from factors that render paper uniquely amenable to such modifications. Firstly, the inherent porosity of paper facilitates facile integration of hydrophobic materials or coatings, thereby augmenting its hydrophobic properties [68]. Secondly, the surface roughness of paper can be finely controlled or tailored to exert a profound influence on its hydrophobic characteristics. To date, several methods have been developed to create hydrophobic paper surfaces using various physical and chemical techniques [69,70]. These techniques include plasma treatment [71], construction of micro-structured surfaces using micro-sized CaCO_3_ and fatty acid [72], rapid extension of supercritical CO_2_ containing alkyl ketene dimer (AKD) through spraying [73], chemical vapor deposition (CVD) of silica particles and polymers [74], dip-coating with AKD [75], and surface-coating by grafting polymers [76]. By implementing these techniques, it is possible to modify the wettability of paper substrates and hence to achieve the desired level of hydrophobicity. This enables the effective preconcentration of microbial cells from urine samples through the process of microbial adhesion.

Based on the well-established observation that microbes exhibit a higher affinity towards different hydrophobic substrates, we propose a hypothesis that the microbes would attach in a similar fashion to paper-based devices. By preconcentrating microbial cells in urine onto hydrophobic paper surfaces, a greater number of target cells will be adhered, leading to a higher concentration of nucleic acids for amplification. This in turn is expected to enhance the overall efficiency of the LAMP assay. In contrast to conventional methods employed to increase LAMP sensitivity, which often involve complex and time-consuming procedures, the preconcentration of microbes onto hydrophobic paper substrates offers a simpler and more cost-effective alternative. By focusing on the innate affinity of microbial cells for hydrophobic surfaces, selective and efficient cell capture can be achieved. Moreover, the reduced reliance on complex equipment and the elimination of laborious manual steps makes this approach more user-friendly and accessible for molecular diagnostics, particularly in resource-limited or remote settings. The proposed approach also holds significant implications for the specific colorimetric detection of pathogens in urine samples [77,78], highlighting its importance in advancing diagnostic capabilities for timely and accurate identification of microbial infections. The simplicity and cost-effectiveness of colorimetric methods make them highly suitable for resource-limited environments and field applications [79].

Furthermore, the approach can be effectively implemented in paper origami-based devices for rapid and onsite nucleic acid detection by integrating key components such as cell adhesion, cell lysis, amplification, and signal readout. By incorporating cell adhesion mechanisms into the design, the paper device allows for the targeted capture and concentration of pathogenic cells. This preconcentration step enhances the sensitivity of the assay by ensuring a higher concentration of the target cells. A myriad of approaches has been investigated by various researchers to perform a LAMP reaction after the initial step of microbial adherence onto paper devices. One such study by Trieu et al. reported the colorimetric identification of live cells based on a nucleic acid amplification testing (NAAT) methodology using an all-in-one origami paper microdevice integrated with DNA purification, loop-mediated isothermal amplification (LAMP), and on-site colorimetric detection [80]. The designed origami microdevice was fabricated using cellulose paper and was composed of five layers including the splitting pad, wicking pad, purification pad, reaction pad, and dye pad. Firstly, the splitting pad was folded towards the purification pad, and the wicking pad was folded underneath the purification pad. Next, a pretreated bacterial solution was introduced into the inlet on the posterior side of the splitting pad, allowing distribution into paper discs on the purification pad. Purification functionality was implemented into the microdevice using chitosan to electrostatically capture DNA. Following the washing steps, the bounded DNA on the purification pad was made ready for amplification without additional elution steps. Subsequently, LAMP reagents were dispensed onto four chambers of the reaction pad. Folding the purification pad on top of the reaction pad exposed the purified DNA to the LAMP reagent solution. The device was then incubated at 65 °C for 30 min to initiate the LAMP reaction. For the visual detection of the LAMP reaction, bleaching solution was introduced into the chambers on the reaction pads and the dye pad (containing methylene blue), which was then folded toward the reaction pad. The blue color on the dye pad showed the presence of live targets [80]. Xu et al. demonstrated the multiplexed determination of microbial species from whole blood through the innovative application of the origami paper-folding technique [81]. This technique facilitated a sequential process including DNA extraction, loop-mediated isothermal amplification (LAMP), and array-based fluorescence detection. The methodology employed in this study involves the dispensing of samples onto the extraction pad preloaded with lysis buffer. Subsequently, the extraction pad was folded onto the LAMP spots, where the loop-mediated isothermal amplification (LAMP) reaction takes place. The resulting DNA amplicons or reaction products were then detected through an array-based fluorescence, providing a visual confirmation of the obtained results [81].

The integration of amplification directly within the paper device streamlines the workflow and eliminates the need for additional equipment. Finally, the signal readout component of the device allows for the interpretation and analysis of the amplified signals, enabling rapid and onsite nucleic acid detection. An artificial intelligence (AI) powered smart-phone app with a machine learning algorithm captures and analyzes the output signals generated by the nucleic acid samples in real-time, providing accurate and reliable results while identifying and rectifying the sources of interference. Thus, by combining the benefits derived from cell adhesion-based preconcentration, LAMP amplification, and user-friendly AI-based signal readout mechanisms within a paper origami-based device, a resilient and portable platform is anticipated to be established. This platform holds immense potential for facilitating sensitive and rapid nucleic acid detection across diverse applications, such as point-of-care diagnostics and field surveillance.

## 3. LAMP Limitations

In the initial phase of adhesion, there is a possibility for capturing non-target microbes. This is because when two different pathogens share similar hydrophobicity and diffusion coefficients, they typically exhibit similar adherence patterns to hydrophobic paper substrates.

Small variations in the fabrication of hydrophobic paper surfaces, or the surface properties of fabricated hydrophobic paper, may lead to inconsistencies in microbial adhesion and preconcentration efficiency. This would affect the reproducibility of results between different batches or experiments.

There can be various other factors apart from the cell surface hydrophobicity (CSH) which could impact the adhesion of the cells to a particular surface. The impact of these confounding factors also needs to be considered. The rate of adhesion can differ depending on the culture conditions of the sample, and hence, reproducibility of the results might be an issue.

Due to limited research in the field of microbial adhesion onto hydrophobic paper adsorbents, there may be significant aspects of the topic that have not been sufficiently addressed in this manuscript. Consequently, it is essential to conduct experimental verification of the hypothesis in future studies to ensure its validity in real-world settings.

## 4. Consequence of the Hypotheses and Discussion

Our hypothesis investigates the potential of preconcentrating microbial cells from urine onto hydrophobic paper surfaces as a promising approach to enhance the efficiency of LAMP amplification. Through the innate affinity of microbial cells for hydrophobic substrates, a higher concentration of target cells is adhered, leading to an increased concentration of nucleic acids for amplification. This preconcentration approach on hydrophobic paper substrates presents several advantages over conventional methods, including simplicity, cost-effectiveness, and suitability for resource-limited settings. By integrating cell adhesion-based preconcentration, LAMP amplification, and user-friendly AI-based signal readout within a paper origami-based device, a robust and portable platform can be established for sensitive and rapid nucleic acid detection. This innovative approach holds significant potential for various applications, ranging from point-of-care diagnostics to field surveillance, and represents a major stride towards advancing molecular diagnostics in diverse settings. Further research and development in this area will undoubtedly contribute to the refinement and widespread implementation of this technology, ultimately benefiting healthcare systems and improving public health outcomes.

## Figures and Tables

**Table 1:** Name of pathogens in urine and their favorable surface hydrophobicities for adhesion.

Sl. No	Type of Pathogen	Name of Pathogen	Favorable Surface	References
1	Gram negative	*E. coli*	Hydrophobic	[58]
2	Gram negative	*Klebsiella pneumoniae*	Hydrophobic	[59]
3	Gram negative	*Pseudomonas aeruginosa*	Hydrophobic	[58]
4	Gram negative	*Proteus mirabilis*	Hydrophobic	
5	Gram positive	*Staphylococcus aureus*	Hydrophobic and Hydrophilic	[50,60,61]
6	Gram positive	*Listeria monocytogenes*	Hydrophobic	[58]
7	Yeast	*Candida albicans*	Hydrophobic	[62]

**Table 2:** Existing literature on microbial adhesion to various hydrophobic material surfaces.

Sl. No	Hydrophobic Material	Pathogen	Detection Method	References
1	Polymeric substrate film	*S. aureus* and *E. coli*	Fluorescence assay with green fluorescent protein (GFP) and bright field microscopy	[31]
2	Knitted polypropylene (PP) and poly-4-hydroxybutyrate (P4HB)	*S. aureus* and *E. coli*	Scanning electron microscopy (SEM)	[63]
3	Titanium dioxide (TiO_2_) surface	*S. epidermidis*	Fluorescence	[64]
4	Plastic surface	*C. albicans*	Hemacytometer measurement	[65]
5	Silane surface	Two strains of *E. coli*, JM109 and D21 and two strains of *Burkholderia cepacia*, G4 and Env435	Column adhesion tests	[66]
6	Hydrophobic Steel Surface	*E. coli*	Scanning electron microscopy (SEM)	[55]

**Table 3:** Influence of surface material on bacterial adhesion and development of biofilm.

Sl. No	Microorganism	Surface Material	Influence on Adhesion and Biofilm Formation	References
1	*E. coli*	Sheets of polyethylene modified by RIGP	Higher bacterial adhesion on positively charged substance. Dense, homogenous, and uniform biofilm formed.	[30]
2	*E. coli*	Layer by layer deposit of cationic polyvinylamine/anionic cellulose nanofibril	Bacterial adhesion and viability increased with increase in surface charge	[30]
3	*S. aureus* and *E. coli*	Polyethylenimine multilayers	Shown to reduce the bacterial adhesion in case of negatively charged surfaces	[30]
4	*S. aureus* and *E. coli*	Gold coated plates with thiol layers	Increases bacterial adhesion as well as biofilm thickness for surfaces, which are hydrophilic and positively charged	[30]
5	*Staphylococcus mutans*	Chimaeric peptide-mediated nanocomplexes of carboxymethyl chitosan or amorphous calcium phosphate	Shows reduced bacterial adhesion for positively charged substances	[30]

## Data Availability

No additional supporting data available.

## References

[1] Sender R., Fuchs S., Milo R. (2016). Revised Estimates for the Number of Human and Bacteria Cells in the Body. PLoS Biol..

[2] Hou K., Wu Z.-X., Chen X.-Y., Wang J.-Q., Zhang D., Xiao C., Zhu D., Koya J.B., Wei L., Li J. (2022). Microbiota in Health and Diseases. Signal Transduct. Target. Ther..

[3] McLellan L.K., Hunstad D.A. (2016). Urinary Tract Infection: Pathogenesis and Outlook. Trends Mol. Med..

[4] Hasandka A., Singh A.R., Prabhu A., Singhal H.R., Nandagopal M.S.G., Mani N.K. (2022). Paper and Thread as Media for the Frugal Detection of Urinary Tract Infections (UTIs). Anal. Bioanal. Chem..

[5] Adrover-Jaume C., Rojo-Molinero E., Clemente A., Russell S.M., Arranz J., Oliver A., De La Rica R. (2020). Mobile Origami Immunosensors for the Rapid Detection of Urinary Tract Infections. Analyst.

[6] Simmering J.E., Tang F., Cavanaugh J.E., Polgreen L.A., Polgreen P.M. (2017). The Increase in Hospitalizations for Urinary Tract Infections and the Associated Costs in the United States, 1998–2011. Open Forum Infect. Dis..

[7] Flores-Mireles A.L., Walker J.N., Caparon M., Hultgren S.J. (2015). Urinary Tract Infections: Epidemiology, Mechanisms of Infection and Treatment Options. Nat. Rev. Microbiol..

[8] Marcus N., Ashkenazi S., Yaari A., Samra Z., Livni G. (2005). Non-*Escherichia coli* versus *Escherichia coli* Community-Acquired Urinary Tract Infections in Children Hospitalized in a Tertiary Center: Relative Frequency, Risk Factors, Antimicrobial Resistance and Outcome. Pediatr. Infect. Dis. J..

[9] Sabih A., Leslie S.W. (2023). Complicated Urinary Tract Infections.

[10] Torres-Sangiao E., Lamas Rodriguez B., Cea Pájaro M., Carracedo Montero R., Parajó Pazos N., García-Riestra C. (2022). Direct Urine Resistance Detection Using VITEK 2. Antibiotics.

[11] Huang B., Zhang L., Zhang W., Liao K., Zhang S., Zhang Z., Ma X., Chen J., Zhang X., Qu P. (2017). Direct Detection and Identification of Bacterial Pathogens from Urine with Optimized Specimen Processing and Enhanced Testing Algorithm. J. Clin. Microbiol..

[12] Sher M., Zhuang R., Demirci U., Asghar W. (2017). Paper-Based Analytical Devices for Clinical Diagnosis: Recent Advances in the Fabrication Techniques and Sensing Mechanisms. Expert Rev. Mol. Diagn..

[13] Sudarsan S., Prabhu A., Prasad D., Mani N.K. (2023). DNA Compaction Enhances the Sensitivity of Fluorescence-Based Nucleic Acid Assays: A Game Changer in Point of Care Sensors?. Analyst.

[14] Kelkar N., Prabhu A., Prabhu A., Nandagopal M.G., Mani N.K. (2022). Sensing of Body Fluid Hormones Using Paper-Based Analytical Devices. Microchem. J..

[15] Hasandka A., Prabhu A., Prabhu A., Singhal H.R., Nandagopal M.S.G., Shenoy R., Mani N.K. (2021). “Scratch It out”: Carbon Copy Based Paper Devices for Microbial Assays and Liver Disease Diagnosis. Anal. Methods.

[16] Prabhu A., Singhal H., Giri Nandagopal M.S., Kulal R., Peralam Yegneswaran P., Mani N.K. (2021). Knitting Thread Devices: Detecting *Candida albicans* Using Napkins and Tampons. ACS Omega.

[17] Singhal H.R., Prabhu A., Giri Nandagopal M.S., Dheivasigamani T., Mani N.K. (2021). One-Dollar Microfluidic Paper-Based Analytical Devices: Do-It-Yourself Approaches. Microchem. J..

[18] Prabhu A., Nandagopal M.S.G., Peralam Yegneswaran P., Prabhu V., Verma U., Mani N.K. (2020). Thread Integrated Smart-Phone Imaging Facilitates Early Turning Point Colorimetric Assay for Microbes. RSC Adv..

[19] Prabhu A., Giri Nandagopal M.S., Peralam Yegneswaran P., Singhal H.R., Mani N.K. (2020). Inkjet Printing of Paraffin on Paper Allows Low-Cost Point-of-Care Diagnostics for Pathogenic Fungi. Cellulose.

[20] Mani N.K., Prabhu A., Biswas S.K., Chakraborty S. (2019). Fabricating Paper Based Devices Using Correction Pens. Sci. Rep..

[21] Ray R., Goyal A., Prabhu A., Parekkh S., Maddasani S., Mani N.K. (2023). Paper-Based Dots and Smartphone for Detecting Counterfeit Country Eggs. Food Chem..

[22] Ray R., Noronha C., Prabhu A., Mani N.K. (2022). Latex-Based Paper Devices with Super Solvent Resistance for On-the-Spot Detection of Metanil Yellow in Food Samples. Food Anal. Methods.

[23] Ray R., Prabhu A., Prasad D., Garlapati V.K., Aminabhavi T.M., Mani N.K., Simal-Gandara J. (2022). Paper-Based Microfluidic Devices for Food Adulterants: Cost-Effective Technological Monitoring Systems. Food Chem..

[24] Campbell J.M., Balhoff J.B., Landwehr G.M., Rahman S.M., Vaithiyanathan M., Melvin A.T. (2018). Microfluidic and Paper-Based Devices for Disease Detection and Diagnostic Research. Int. J. Mol. Sci..

[25] Bhattarai R.K., Pudasaini S., Sah M., Neupane B.B., Giri B. (2022). Handmade Paper as a Paper Analytical Device for Determining the Quality of an Antidiabetic Drug. ACS Omega.

[26] Martinez A.W., Phillips S.T., Whitesides G.M., Carrilho E. (2010). Diagnostics for the Developing World: Microfluidic Paper-Based Analytical Devices. Anal. Chem..

[27] St John A., Price C.P. (2014). Existing and Emerging Technologies for Point-of-Care Testing. Clin. Biochem. Rev..

[28] Notomi T., Mori Y., Tomita N., Kanda H. (2015). Loop-Mediated Isothermal Amplification (LAMP): Principle, Features, and Future Prospects. J. Microbiol..

[29] Özay B., McCalla S.E. (2021). A Review of Reaction Enhancement Strategies for Isothermal Nucleic Acid Amplification Reactions. Sens. Actuators Rep..

[30] Zheng S., Bawazir M., Dhall A., Kim H.-E., He L., Heo J., Hwang G. (2021). Implication of Surface Properties, Bacterial Motility, and Hydrodynamic Conditions on Bacterial Surface Sensing and Their Initial Adhesion. Front. Bioeng. Biotechnol..

[31] Yuan Y., Hays M.P., Hardwidge P.R., Kim J. (2017). Surface Characteristics Influencing Bacterial Adhesion to Polymeric Substrates. RSC Adv..

[32] Saito Y., Nakagami K., Hempel G. (2020). Chapter 1—Sample Preparation for the Analysis of Drugs in Biological Fluids. Methods of Therapeutic Drug Monitoring Including Pharmacogenetics.

[33] Hagarová I. (2017). Cloud Point Extraction Utilizable for Separation and Preconcentration of (Ultra)Trace Elements in Biological Fluids before Their Determination by Spectrometric Methods: A Brief Review. Chem. Pap..

[34] Chinnappan R., Ramadan Q., Zourob M. (2023). Isolation and Detection of Exosomal Mir210 Using Carbon Nanomaterial-Coated Magnetic Beads. J. Funct. Biomater..

[35] Chinnappan R., Ramadan Q., Zourob M. (2023). An Integrated Lab-on-a-Chip Platform for Pre-Concentration and Detection of Colorectal Cancer Exosomes Using Anti-CD63 Aptamer as a Recognition Element. Biosens. Bioelectron..

[36] Bouatra S., Aziat F., Mandal R., Guo A.C., Wilson M.R., Knox C., Bjorndahl T.C., Krishnamurthy R., Saleem F., Liu P. (2013). The Human Urine Metabolome. PLoS ONE.

[37] Ridley J.W., Ridley J.W. (2018). Metabolic Origins of Urine and Other Body Fluids BT. Fundamentals of the Study of Urine and Body Fluids.

[38] Zuo Y., Yang Y., Zhu Z., He W., Aydin Z. (2011). Determination of Uric Acid and Creatinine in Human Urine Using Hydrophilic Interaction Chromatography. Talanta.

[39] Fritsch J., Sankey O.F., Schmidt K.E., Page J.B. (1999). Chemical Reactions of Ammonia with Polar and Non-Polar Nitride Semiconductor Surfaces. Surf. Sci..

[40] Lu H.D., Yang S.S., Wilson B.K., McManus S.A., Chen C.V.H.H., Prud’homme R.K. (2017). Nanoparticle Targeting of Gram-Positive and Gram-Negative Bacteria for Magnetic-Based Separations of Bacterial Pathogens. Appl. Nanosci..

[41] Jaggessar A., Shahali H., Mathew A., Yarlagadda P.K.D. (2017). V Bio-Mimicking Nano and Micro-Structured Surface Fabrication for Antibacterial Properties in Medical Implants. J. Nanobiotechnol..

[42] Palmer J., Flint S., Brooks J. (2007). Bacterial Cell Attachment, the Beginning of a Biofilm. J. Ind. Microbiol. Biotechnol..

[43] Gottenbos B., Van Der Mei H.C., Busscher H.J., Grijpma D.W., Feijen J. (1999). Initial Adhesion and Surface Growth of *Pseudomonas Aeruginosa* on Negatively and Positively Charged Poly(Methacrylates). J. Mater. Sci. Mater. Med..

[44] Yoda I., Koseki H., Tomita M., Shida T., Horiuchi H., Sakoda H., Osaki M. (2014). Effect of Surface Roughness of Biomaterials on *Staphylococcus Epidermidis* Adhesion. BMC Microbiol..

[45] James G.A., Boegli L., Hancock J., Bowersock L., Parker A., Kinney B.M. (2019). Bacterial Adhesion and Biofilm Formation on Textured Breast Implant Shell Materials. Aesthetic Plast. Surg..

[46] Kolewe K.W., Peyton S.R., Schiffman J.D. (2015). Fewer Bacteria Adhere to Softer Hydrogels. ACS Appl. Mater. Interfaces.

[47] Zita A., Hermansson M. (1997). Determination of Bacterial Cell Surface Hydrophobicity of Single Cells in Cultures and in Wastewater in Situ. FEMS Microbiol. Lett..

[48] Krasowska A., Sigler K. (2014). How Microorganisms Use Hydrophobicity and What Does This Mean for Human Needs?. Front. Cell. Infect. Microbiol..

[49] Oh J.K., Yegin Y., Yang F., Zhang M., Li J., Huang S., Verkhoturov S.V., Schweikert E.A., Perez-Lewis K., Scholar E.A. (2018). The Influence of Surface Chemistry on the Kinetics and Thermodynamics of Bacterial Adhesion. Sci. Rep..

[50] Tegoulia V.A., Cooper S.L. (2002). *Staphylococcus aureus* Adhesion to Self-Assembled Monolayers: Effect of Surface Chemistry and Fibrinogen Presence. Colloids Surf. B Biointerfaces.

[51] Kim Y., Jung K., Chang J., Kwak T., Lim Y., Kim S., Na J., Lee J., Choi I., Lee L.P. (2019). Active Surface Hydrophobicity Switching and Dynamic Interfacial Trapping of Microbial Cells by Metal Nanoparticles for Preconcentration and In-Plane Optical Detection. Nano Lett..

[52] Mi F., Hu C., Wang Y., Wang L., Peng F., Geng P., Guan M. (2022). Recent Advancements in Microfluidic Chip Biosensor Detection of Foodborne Pathogenic Bacteria: A Review. Anal. Bioanal. Chem..

[53] Hizal F., Rungraeng N., Lee J., Jun S., Busscher H.J., van der Mei H.C., Choi C.-H. (2017). Nanoengineered Superhydrophobic Surfaces of Aluminum with Extremely Low Bacterial Adhesivity. ACS Appl. Mater. Interfaces.

[54] Li X., Zhang Y., Gulbins E., Da Silva M. (2010). Handbook of Hydrocarbon and Lipid Microbiology.

[55] Wosten H.A.B., De Vries O.M.H., Wessels J.G.H. (1993). Interfacial Self-Assembly of a Fungal Hydrophobin into a Hydrophobic Rodlet Layer. Plant Cell.

[56] Bayry J., Aimanianda V., Guijarro J.I., Sunde M., Latgé J.-P. (2012). Hydrophobins–Unique Fungal Proteins. PLoS Pathog..

[57] Masuoka J., Hazen K.C. (1997). Cell Wall Protein Mannosylation Determines *Candida Albicans* Cell Surface Hydrophobicity. Microbiology.

[58] Farniya F., Jamalli A., Dadgar T. (2019). Physicochemical Surface Characteristics in Different Pathogenic Bacteria. Cogent Biol..

[59] Meno Y., Amako K. (1991). The Surface Hydrophobicity and Avirulent Character of an Encapsulated Strain of *Klebsiella pneumoniae*. Microbiol. Immunol..

[60] Maikranz E., Spengler C., Thewes N., Thewes A., Nolle F., Jung P., Bischoff M., Santen L., Jacobs K. (2020). Different Binding Mechanisms of: *Staphylococcus aureus* to Hydrophobic and Hydrophilic Surfaces. Nanoscale.

[61] Reifsteck F., Wee S., Wilkinson B.J. (1987). Hydrophobicity-Hydrophilicity of Staphylococci. J. Med. Microbiol..

[62] Ellepola A.N.B., Samaranayake L.P. (2001). Investigative Methods for Studying the Adhesion and Cell Surface Hydrophobicity of Candida Species: An Overview. Microb. Ecol. Health Dis..

[63] Verhorstert K.W., Guler Z., de Boer L., Riool M., Roovers J.-P.W., Zaat S.A. (2020). In Vitro Bacterial Adhesion and Biofilm Formation on Fully Absorbable Poly-4-Hydroxybutyrate and Nonabsorbable Polypropylene Pelvic Floor Implants. ACS Appl. Mater. Interfaces.

[64] Wassmann T., Kreis S., Behr M., Buergers R. (2017). The Influence of Surface Texture and Wettability on Initial Bacterial Adhesion on Titanium and Zirconium Oxide Dental Implants. Int. J. Implant Dent..

[65] Klotz S.A., Drutz D.J., Zajic J.E. (1985). Factors Governing Adherence of Candida Species to Plastic Surfaces. Infect. Immun..

[66] Salerno M.B., Logan B.E., Velegol D. (2004). Importance of Molecular Details in Predicting Bacterial Adhesion to Hydrophobic Surfaces. Langmuir.

[67] Kim H.T., Jung S.K., Kim D.-E., Park C.Y., Lee S.-Y. (2022). Wettability Control of Paper through Substitution between the Hydroxyl Group and Carbon Elements Using Argon-Carbon Plasma Treatment. Vacuum.

[68] Modaressi H., Garnier G. (2002). Mechanism of Wetting and Absorption of Water Droplets on Sized Paper: Effects of Chemical and Physical Heterogeneity. Langmuir.

[69] Wen Q., Guo F., Yang F., Guo Z. (2017). Green Fabrication of Coloured Superhydrophobic Paper from Native Cotton Cellulose. J. Colloid Interface Sci..

[70] Baidya A., Ganayee M.A., Jakka Ravindran S., Tam K.C., Das S.K., Ras R.H.A., Pradeep T. (2017). Organic Solvent-Free Fabrication of Durable and Multifunctional Superhydrophobic Paper from Waterborne Fluorinated Cellulose Nanofiber Building Blocks. Acs Nano.

[71] Balu B., Kim J.S., Breedveld V., Hess D.W. (2009). Tunability of the Adhesion of Water Drops on a Superhydrophobic Paper Surface via Selective Plasma Etching. J. Adhes. Sci. Technol..

[72] Hu Z., Zen X., Gong J., Deng Y. (2009). Water Resistance Improvement of Paper by Superhydrophobic Modification with Microsized CaCO_3_ and Fatty Acid Coating. Colloids Surf. A Physicochem. Eng. Asp..

[73] Werner O., Quan C., Turner C., Pettersson B., Wågberg L. (2010). Properties of Superhydrophobic Paper Treated with Rapid Expansion of Supercritical CO_2_ Containing a Crystallizing Wax. Cellulose.

[74] Yang H., Deng Y. (2008). Preparation and Physical Properties of Superhydrophobic Papers. J. Colloid Interface Sci..

[75] Arbatan T., Zhang L., Fang X.-Y., Shen W. (2012). Cellulose Nanofibers as Binder for Fabrication of Superhydrophobic Paper. Chem. Eng. J..

[76] Carlmark A., Malmström E.E. (2003). ATRP Grafting from Cellulose Fibers to Create Block-Copolymer Grafts. Biomacromolecules.

[77] David S., Munteanu R.-E., Tițoiu A.-M., Petcu I.-C., Cernat I.-C., Leancu C., Gheorghiu M., Gheorghiu E. (2022). Direct, Rapid Detection of Pathogens from Urine Samples. Materials.

[78] Wallis C., Melnick J.L., Longoria C.J. (1981). Colorimetric Method for Rapid Determination of Bacteriuria. J. Clin. Microbiol..

[79] Qian S., Cui Y., Cai Z., Li L. (2022). Applications of Smartphone-Based Colorimetric Biosensors. Biosens. Bioelectron. X.

[80] Trieu P.T., Lee N.Y. (2019). Paper-Based All-in-One Origami Microdevice for Nucleic Acid Amplification Testing for Rapid Colorimetric Identification of Live Cells for Point-of-Care Testing. Anal. Chem..

[81] Xu G., Nolder D., Reboud J., Oguike M.C., van Schalkwyk D.A., Sutherland C.J., Cooper J.M. (2016). Paper-Origami-Based Multiplexed Malaria Diagnostics from Whole Blood. Angew. Chem. Int. Ed..

